# Books and Magazines

**Published:** 1902-02

**Authors:** 


Peru: History of Coca, "The Divine Plant" of the Incas, with an introductory account of the incas, and of the Andean Indians of today. By W. Golden Mortimer, M. D. With one hundred and seventy-eight illustrations. Price, $5.00, net. New York: J. H. Vail & Company. 1901.

This is by far the most exhaustive work on the subject that has yet been written.

It answers every problem on the use of the divine plant of the Incas and gives the first scientific explanation of the application of Coca to the production of energy and endurance as a means to increase man's period of usefulness and consequent happiness.

Completed after years of careful investigation. The thoroughness of research may be appreciated from the contained bibliography of nearly 600 titles.

The book embraces on new lines an abundance of food for thought presented in a manner ready for assimilation. Travel, adventure, antiquities, conquest, history and the philosophy of living are so cleverly blended with the scientific facts of the subject treated that no matter what may be one's occupation or hobby, the book will be found to be impressively entertaining to the reader.

The whole rendered available for reference by an exhaustive index covering thirty-two double column pages.

Six hundred and eight pages, octavo, on heavy paper, printed from new type, profusely illustrated, handsomely bound in cloth, beveled edges, gilt top.

WARWICK OF THE KNOBS.—[We loaned this book to a young lady. Here is her "review."—ED.]

One of the numerous candidates for favor among the books for 1901 is "Warwick of the Knobs," by John Uri Lloyd. The scene is laid in Kentucky, and the time is during the late war between the States. The hero is the elder Warwick, a "hard-shelled" Baptist preacher, a typical representative of that religious sect. He was as iron-clad in his politics as in his religion. In neither, in the delineation of the characters, is there anything new to the people, or original to the author. The same thoughts are put into Warwick's head that have been expressed in print, time and again, for thirty years. Whether true or false, does not concern us. Suffice it to say, that he was intensely Southern in his political views and orthodox with his people in theology.

The sentiment of the book will be approved in the South, and condemned in the North. The universal opinion will be that it is not a "long felt want." When Warwick rescued Lionel, the fossil-hunter and rock-smasher, from the flood and took him to his home, he did what his religion taught him, and what any philanthropic person would have done. That Mary Warwick should fall in love with the young collegian and be induced to elope and then be deceived is nothing new. This incident is as old as the wagon load of fossils that Joshua hauled to Covington for the rock hunter.

It seems that Joshua, the son, was the only philosopher in the whole story, but he is stripped of the characteristics of a true Southron. The author, to have portrayed Southern character truly, should have made Joshua perforate the body of Lionel with a bullet, and not to have allowed himself to be dazed by a few imaginary pale-faces, and then permitted the villian to escape. The triumph, if there is any, is with vice and wrong. One does not get up refreshed and invigorated after reading the book—a tired sort of feeling steals o'er the reader, and he feels disgusted with the way the story ends.

The pictures are, almost without exception, interesting and clearly executed. The publishers have shown a most commendable enterprise in the mechanical work attending the production of the volume.

